# 混合型离子交换液相色谱-串联质谱法检测鸡蛋中10种氨基糖苷类药物残留

**DOI:** 10.3724/SP.J.1123.2021.02027

**Published:** 2021-12-08

**Authors:** Lili WEI, Xia XUE, Chuanxiang WU, Yi DING, Lanxiang LU, Jun WANG, Yanming LIU

**Affiliations:** 1.山东省食品药品检验研究院, 山东 济南 250101; 1. Shandong Institute for Food and Drug Control, Jinan 250101, China; 2.山东省食品药品安全检测工程技术研究中心, 山东 济南 250101; 2. Shandong Research Center of Engineering and Technology for Safety Inspection of Food and Drug, Jinan 250101, China

**Keywords:** 固相萃取, 混合型离子交换液相色谱, 串联质谱, 氨基糖苷类药物, 鸡蛋, solid phase extraction (SPE), mixed-mode ion exchange liquid chromatography, tandem mass spectrometry (MS/MS), aminoglycosides, eggs

## Abstract

该研究系统地优化了样品前处理过程及仪器分析中影响氨基糖苷残留分析准确度与灵敏度的各主要因素,建立了鸡蛋中10种氨基糖苷类药物(链霉素、双氢链霉素、潮霉素B、卡那霉素、阿米卡星、妥布霉素、安普霉素、大观霉素、新霉素、庆大霉素)残留量的混合型离子交换液相色谱-串联质谱分析方法。样品经10 mmol/L乙酸铵缓冲溶液(含0.4 mmol/L EDTA和50 g/L三氯乙酸)超声提取,调节pH至6~7后,经PRiME HLB固相萃取柱富集净化,采用SIELC Obelisc R色谱柱分离,以乙腈和1.0%(v/v)甲酸水溶液(含1 mmol/L甲酸铵)为流动相进行梯度洗脱,在正离子、多反应监测模式下经串联质谱仪测定,外标法定量。该方法在5~200 μg/L质量浓度范围内线性关系良好,相关系数(*r*^2^)均大于0.99;方法的检出限(LOD, *S/N*≥3)为2~5 μg/kg,定量限(LOQ, *S/N*≥10)为5~10 μg/kg。在空白鸡蛋中进行LOQ、20 μg/kg、100 μg/kg 3个水平的加标回收实验,方法的平均回收率(*n*=6)为68.1%~111.3%,相对标准偏差为1.2%~12.3%。利用该方法对市售的20批次鸡蛋样品进行测定,均未检出目标物。本方法简单、灵敏、准确,可实现鸡蛋中10种氨基糖苷类药物残留的批量检测。

## Abstract (en)

Aminoglycosides (AGs) are a class of broad spectrum antibiotics that have bactericidal activity against some aerobic gram-positive and gram-negative organisms. AGs have been extensively employed in animal husbandry for the treatment of bacterial infections or growth promotion. Many countries have issued strict maximum residue levels (MRLs) for AGs in many animal-origin foods. Analysis of AGs is quite challenging due to their physicochemical properties. The lack of any notable chromophores or fluorophores makes direct detection using ultraviolet (UV) or fluorescence (FLR) spectroscopy unfeasible. Therefore, AGs must be derivatized before they can be analyzed by UV or FLR detection techniques. However, the sensitivity of such derivatization methods is relatively low. Methods based on chromatographic analysis coupled with tandem mass spectrometric detection are emerging as the most common way of identification and quantification. The retention of AGs on reversed-phase column is poor due to the presence of various amino and hydroxyl groups in their structures. Therefore, ion-pair chromatography has reportedly been used to improve the retention of AGs. However, electrospray ionization-mass spectrometric detection was hampered by using an ion pairing reagent due to the suppression of ionization. In this study, a method based on mixed-mode ion exchange liquid chromatography-tandem mass spectrometry was developed for the determination of ten AGs residues (streptomycin, dihydrostreptomycin, hygromycin B, kanamycin, amikacin, tobramycin, apramycin, spectinomycin, neomycin, and gentamycin) in eggs. The main factors governing the method, such as the type of chromatographic column used, the type and proportion of the mobile phase used, mass spectroscopy parameters, type and volume of the extraction solvent used, pH, and the type of solid phase extraction (SPE) column, were investigated during sample pretreatment and instrument analysis. The residues of AGs in the test samples were extracted by ultrasonication with 10 mmol/L ammonium acetate buffer solution (comprising 0.4 mmol/L EDTA and 50 g/L trichloroacetic acid). After adjusting the pH, the AG residues in the sample were purified and enriched using a PRiME HLB SPE column. The target analytes were separated on a SIELC Obelisc R column (150 mm×2.1mm, 5 μm), the column temperature being 40 ℃, the flow rate being 0.3 mL/min, and the injection volume being 5 μL. Gradient elution was carried out with acetonitrile and 1.0%(v/v) formic acid aqueous solution (including 1 mmol/L ammonium formate) as the mobile phases. The detection was performed by electrospray ionization-tandem mass spectrometry (ESI-MS/MS) in multiple reaction monitoring (MRM) mode. The retention times and ionic ratios were used for qualitative analysis, and the peak areas were used for quantitative analysis by the external standard method. Good correlation coefficients exceeding 0.99 were observed for all the AGs in the concentration range of 5-200 μg/L under the optimum conditions. The limits of detection (LODs, *S/N* ≥ 3) and limits of quantification (LOQs, *S/N*≥10) for the ten AGs were 2-5 μg/kg and 5-10 μg/kg, respectively. The recoveries ranged from 68.1% to 111.3% (*n*=6) at three levels (LODs, 20 μg/kg, and 100 μg/kg) in spiked blank egg samples, and the relative standard deviations were 1.2%-12.3%. The matrix effects of the analytes were between 0.3% and 94.3% after purification on the PRiME HLB column. The applicability of the method was validated by analyzing egg samples purchased from local markets. Overall, the method of mixed-mode ion exchange liquid chromatography-tandem mass spectrometry has proven to be a reliable and powerful technique for the simultaneous quantification and confirmation of ten AGs without using an ion pair reagent. Moreover, the clean-up step only required a kind of PRiME HLB sorbent cartridge. The relative parameter data of established method were consistent with GB/T 27404-2008. With simple pretreatment, rapid determination and high sensitivity, the method can be used in the determination of AGs in eggs.

氨基糖苷类药物(aminoglycosides, AGs)是一种广谱的抗生素,对革兰氏阴性菌有显著的抗菌效果^[1]^,在畜牧业和养殖业应用广泛。AGs不仅治疗动物疾病,还可以用作饲料添加剂,促进动物的生长发育^[2]^。AGs具有一定的耳毒性、肾毒性和神经肌肉阻滞作用^[3]^,该类抗生素被畜禽动物食用后,主要存在于肝脏、肾脏、肌肉、奶和蛋中,因此可以通过蛋中抗生素残留检测方法的建立,来监测家禽动物食品的质量状况。

世界各国均建立了AGs在禽蛋中的最高残留限量(MRL)。欧盟^[4]^规定鸡蛋中新霉素的MRL为500 μg/kg,产蛋期禁止使用卡那霉素、安普霉素、大观霉素;我国GB 31650-2019^[5]^规定鸡蛋中大观霉素的MRL为2000 μg/kg,新霉素的MRL为500 μg/kg,潮霉素B不得检出。目前文献中对AGs的检测针对的基质多是蜂蜜、乳制品、肉制品、水产品等^[6,7,8,9,10,11,12,13]^,对于鸡蛋中AGs的检测相关报道较少。建立鸡蛋中AGs残留量的快速、准确的检测方法,对维护人类的饮食健康具有重要意义。

目前AGs残留的检测技术已经取得很大进展。酶联免疫法操作相对简单,但无法准确定性,常作为筛查方法^[14]^。气相色谱法需要衍生,步骤繁琐^[15]^;液相色谱法直接测定AGs时需要通用型的检测器,检出限高^[16]^,间接测定时需要对分析物进行柱前或柱后衍生,重现性差^[17]^。液相色谱-串联质谱法由于抗基质干扰能力强、灵敏度高等优点,在AGs的检测方面应用最为广泛。在传统的反相色谱中,需要加入离子对试剂改善AGs的色谱保留行为,但离子对试剂进入质谱后容易产生离子抑制。亲水作用色谱和离子交换色谱由于对极性化合物有很好的保留,与MS/MS联用,可以解决上述问题。

PRiME HLB固相萃取柱能够去除鸡蛋中磷脂、脂肪、蛋白质等常见的基质干扰物,常被用作动物源性食品中多种兽药残留测定的净化手段。目前,未有该固相萃取柱在AGs净化方面的应用。本研究采用PRiME HLB固相萃取柱净化,Obelisc R色谱柱分离,MS/MS检测,实现了鸡蛋中链霉素、双氢链霉素、潮霉素B、卡那霉素、阿米卡星、妥布霉素、安普霉素、大观霉素、新霉素、庆大霉素等10种AGs的同时检测。该方法操作简单、避免了离子对试剂的使用,能够满足国内外对鸡蛋中AGs残留量的检测要求。

## 1 实验部分

### 1.1 仪器、试剂与材料

岛津LC-30AD超高效液相色谱仪,配自动进样器和柱温箱(日本Shimadzu公司), 8050三重四极杆质谱仪,配有电喷雾电离源(ESI)(日本Shimadzu公司); 3-18K台式高速离心机(德国Sigma公司); BT 125D电子天平(德国Sartorius公司); MS3型旋涡混合器(德国IKA公司); SB-800DTD超声波清洗仪(宁波新芝生物科技股份有限公司); Milli-Q超纯水系统(美国Millipore公司);固相萃取装置(美国Supelco公司)。

链霉素(STREP, CAS号:3810-74-0,纯度90.3%)、双氢链霉素(DHSTREP, CAS号:5490-27-7,纯度94.4%)、潮霉素B(HYGRO, CAS号:31282-04-9,纯度90.0%)、阿米卡星(AMIK, CAS号:1257517-67-1,纯度99.0%)、安普霉素(APRA, CAS号:65710-07-8,纯度81.5%)、大观霉素(SPEC, CAS号:22189-32-8,纯度89.4%)、新霉素(NEO)、庆大霉素(GENT, CAS号:1405-41-0,纯度90.8%)(德国Dr. Ehrenstorfer公司);卡那霉素(KANA, CAS号:25389-94-0,纯度97.0%)(北京曼哈格生物科技有限公司);妥布霉素(TOBRA, CAS号:32986-56-4,纯度94.0%)(美国Stanford Chemicals公司);甲醇、乙腈、甲酸(色谱纯,德国Merck公司);甲酸铵(色谱纯,美国Fisher公司);三氯乙酸(TCA)、乙二胺四乙酸二钠(EDTA)、盐酸、氢氧化钠、氨水、乙酸铵(分析纯,国药集团化学试剂有限公司);所用水为超纯水。

Prime HLB固相萃取柱(200 mg, 6 mL)(美国Waters公司)。

### 1.2 标准溶液的配制

标准储备溶液:准确称取适量的10种标准品,分别溶解于10.0 mL水中,配制成质量浓度为1000 mg/L的标准储备液,于4 ℃避光保存。

混合标准中间溶液:取各标准储备溶液,混合,用水逐级稀释,配成质量浓度为10、1和0.1 mg/L的混合标准中间溶液。

试剂标准工作溶液:取5、10、20 μL 1 mg/L的混合标准中间液和5、10、20 μL 10 mg/L的混合标准中间液,用甲酸-乙腈-水(10∶5∶85, v/v/v)定容至1.0 mL,配成5、10、20、50、100、200 μg/L试剂标准工作溶液。

### 1.3 色谱-质谱条件

色谱柱:SIELC Obelisc R (150 mm×2.1 mm, 5 μm,美国SIELC公司);流动相:A相为乙腈溶液,B相为1%(v/v)甲酸溶液(含1 mmol/L甲酸铵);柱温:40 ℃;进样量:5 μL;梯度洗脱程序:0~3.0 min,75%A; 3.0~6.0 min,75%A~20%A; 6.0~8.0 min,20%A~5%A; 8.0~11.0 min,5%A; 11.0~11.1 min,5%A~75%A; 11.1~16.0 min,75%A。

电喷雾离子源(ESI);多反应监测(MRM)模式;正离子扫描方式;雾化气流量:3 L/min;加热气流量:10 L/min;接口温度:300 ℃; 脱溶剂管温度:250 ℃;加热块温度:400 ℃;干燥气流量:10 L/min。10种氨基糖苷类药物的定量离子对、定性离子对、驻留时间、Q1 pre bias、Q3 pre bias见表1。

**表1:** 10种氨基糖苷类药物的MS/MS参数

Compound	Parent ion (m/z)	Daughter ion (m/z)	Dwell time/ms	Q1 pre bias/V	Collision energy/eV	Q3 pre bias/V
Streptomycin (STREP)	582.0	246.0	15	30	36	27
		263.1^*^	15		33	17
Dihydrostreptomycin (DHSTREP)	584.2	246.1	13	30	39	26
		263.1^*^	13		32	28
Tobramycin (TOBRA)	468.0	163.2^*^	15	25	24	17
		324.3	15		16	15
Hygromycin B (HYGRO)	527.9	177.1^*^	20	26	33	18
		352.1	20		25	23
Amikacin (AMIK)	586.0	163.1^*^	13	30	35	10
		425.2	13		21	28
Apramycin (APRA)	540.0	217.2^*^	13	28	31	14
		378.2	13		19	19
Spectinomycin (SPEC)	332.8	112.2	20	17	28	12
		140.1^*^	20		23	14
Neomycin (NEO)	615.2	161.0^*^	25	32	29	10
		293.2	25		27	14
Gentamycin (GENT)	478.0	157.1	25	24	22	17
		322.1^*^	25		15	16
Kanamycin (KANA)	485.1	163.1^*^	13	25	25	17
		324.2	13		19	16

* Quantitative ion.

### 1.4 样品处理

1.4.1 提取

准确称取2.5 g试样(精确至0.01 g)于50 mL离心管中,加入10 mL 10 mmol/L乙酸铵缓冲溶液(含0.4 mmol/L EDTA和50 g/L三氯乙酸)涡旋混匀,超声15 min, 8000 r/min离心5 min,将上清液转移至另一50 mL离心管中,残渣再用10 mL提取液重复提取一次,合并两次的上清液,用10 mol/L氢氧化钠溶液调pH至6.5,用水定容至25 mL。过滤,收集滤液待净化。

1.4.2 净化

依次用3 mL甲醇、3 mL水活化Prime HLB固相萃取柱。准确移取20 mL上清液,以不高于1 mL/min的流速全部通过固相萃取柱,弃去滤液;用3 mL超纯水淋洗两次,3 mL甲醇-水(5∶95, v/v)淋洗一次,弃去淋洗液,将小柱抽干;用2 mL甲酸-乙腈-水(10∶5∶85, v/v/v)溶液洗脱并收集洗脱液,抽干固相萃取柱,涡旋混匀后,经微孔滤膜过滤,供液相色谱-串联质谱仪分析测定。

1.4.3 基质匹配标准工作溶液

取6份空白样品于50 mL离心管中,按1.4.1节和1.4.2节操作,制得空白提取液。分别吸取5、10、20 μL 1 mg/L的混合标准中间溶液和5、10、20 μL 10 mg/L的混合标准中间溶液,用空白提取液定容至1.0 mL,配成5、10、20、50、100、200 μg/L的基质匹配标准工作溶液。

### 1.5 基质效应研究

将基质匹配标准工作溶液和试剂标准工作溶液分别注入超高效液相色谱-串联质谱仪,按1.3节进行测定,得到各组分的峰面积。以峰面积为纵坐标,以标准溶液质量浓度为横坐标绘制标准曲线,以曲线的斜率评价基质效应(ME)。基质效应计算公式^[7]^为:ME=(基质匹配标准曲线的斜率/试剂标准曲线的斜率-1)×100%。

## 2 结果与讨论

### 2.1 提取条件的选择

2.1.1 提取溶剂的选择

由于AGs类药物难溶于甲醇、乙腈等有机溶剂,因此通常采用缓冲液进行提取,同时加入三氯乙酸既可以去除蛋白质,也可以溶解该类药物。结合文献,本研究考察了提取液1(10 mmol/L乙酸铵缓冲液、50 g/L TCA)^[6]^、提取液2(10 mmol/L乙酸铵缓冲液、50 g/L TCA、0.4 mmol/L EDTA)^[18]^和提取液3(10 mmol/L乙酸铵缓冲液、50 g/L TCA、0.4 mmol/L EDTA、5 g/L NaCl)^[19]^ 3种提取溶剂对鸡蛋中氨基糖苷类药物回收率的影响。向空白鸡蛋基质中加入100 μg/kg的标准品,用上述提取溶剂进行提取,基质匹配标准工作曲线进行定量,计算回收率。由图1可以看出,用提取溶剂1和提取溶剂2提取后HYGRO的回收率在73%左右,而用提取溶剂3提取后,HYGRO的回收率为53.2%,表明氯化钠的加入会降低HYGRO的回收率。对实验数据进一步分析时发现用提取液1提取后STREP、DHSTREP和HYGRO的基质效应分别为-79%、-73%和-40%,用提取液2提取后对应的基质效应分别为-0.3%、-12.5%和-12.1%,说明EDTA的加入会减少STREP、DHSTREP和HYGRO的基质效应。综合以上因素,选择提取液2(10 mmol/L乙酸铵缓冲液、50 g/L TCA、0.4 mmol/L EDTA)为提取溶剂。

**图1:**
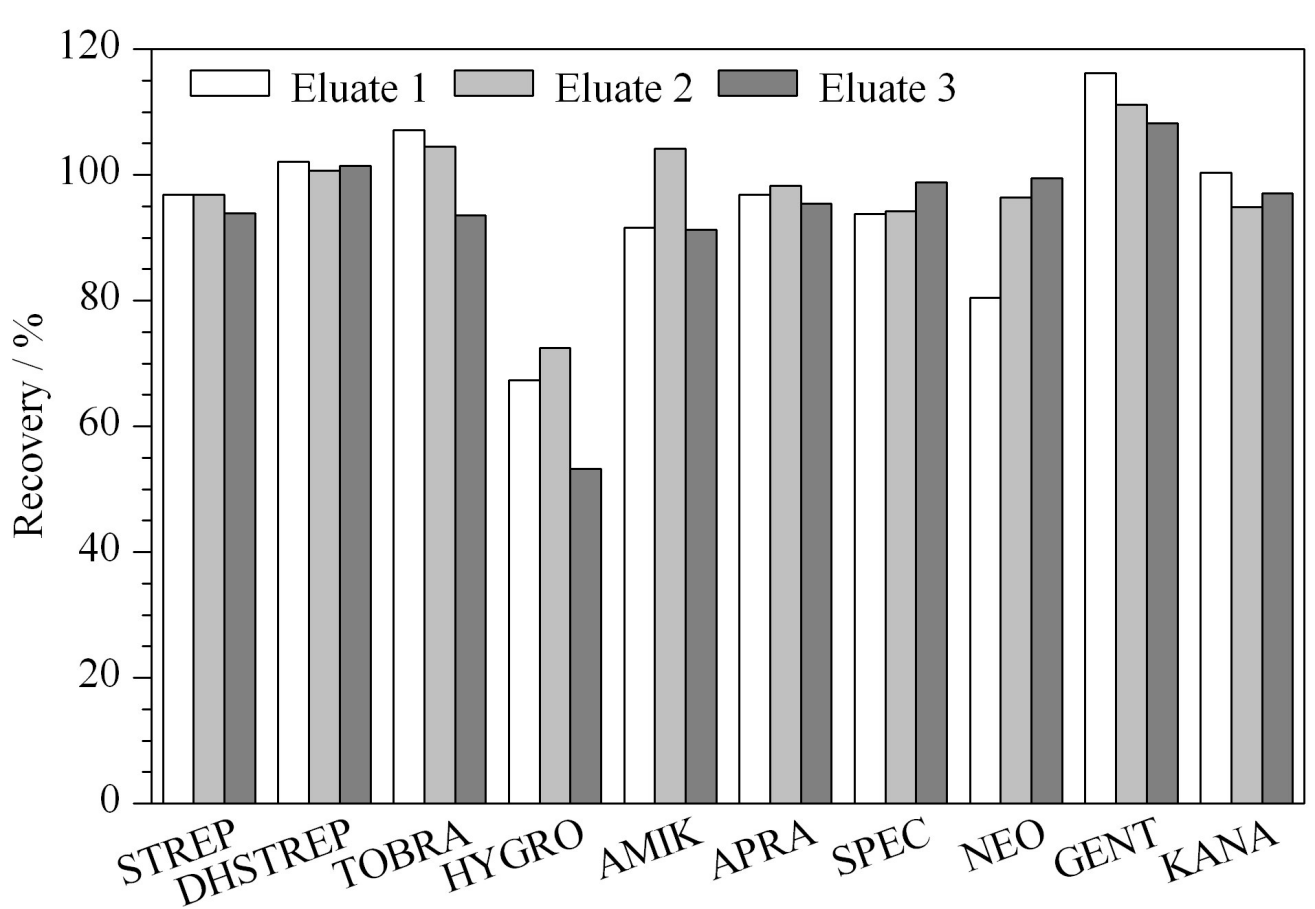
采用不同提取液时鸡蛋中10种氨基糖苷类药物的加标回收率

2.1.2 提取体积和次数的选择

分别用10 mL、5 mL+5 mL、20 mL、10 mL+10 mL、30 mL提取溶剂提取100 μg/kg的空白加标样品,10种氨基糖苷的回收率见图2。对样品只提取1次时,当提取剂的体积达到20 mL时,回收率基本稳定;当用20 mL提取剂分两次提取时,回收率有明显提高,因此,选择提取体积为20 mL,提取次数2次。

**图2:**
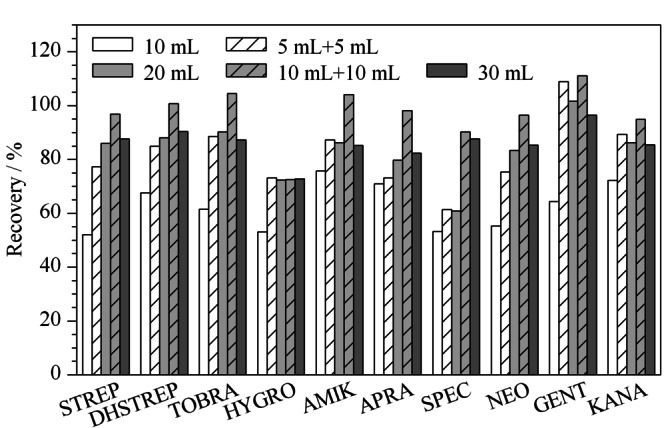
提取体积和提取次数对鸡蛋中10种氨基糖苷类药物加标回收率的影响

### 2.2 净化条件的选择

2.2.1 固相萃取柱的选择

鸡蛋样品中含有丰富的蛋白质、卵磷脂及固醇类物质,干扰分析物的测定,需要对样品进行净化和富集。本研究比较了Oasis PRiME HLB(200 mg/6 mL, Waters)、WCX(60 mg/3 mL, Waters)、MCX (60 mg/3 mL, Waters)和SupelMIP(50 mg/3 mL, Supelco)4种固相萃取柱对10种氨基糖苷回收率的影响(见图3)。

**图3:**
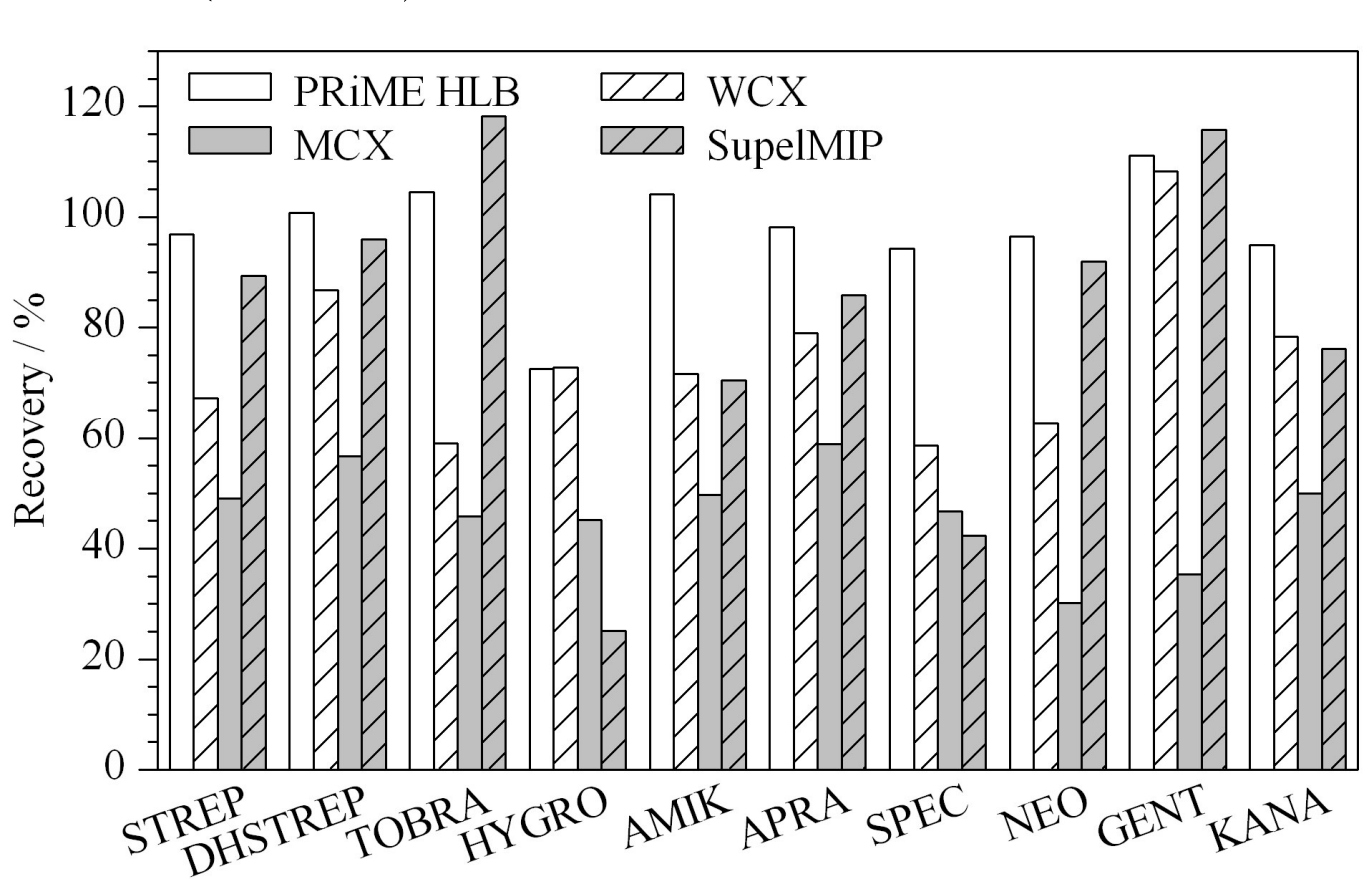
固相萃取柱对鸡蛋中10种氨基糖苷类药物加标回收率的影响

MCX是混合型强阳离子交换柱,与氨基糖苷类药物的作用较强,只用氨水甲醇很难完全洗脱,因此回收率较低;WCX是一种弱阳离子交换柱,与MCX固相萃取柱相比,回收率有所提高,但是大部分化合物的回收率仍低于80%,可能的原因为提取液中三氯乙酸增加了溶液的离子强度,影响了氨基糖苷类药物的保留;SupelMIP是针对氨基糖苷类物质的分子印迹柱,但是对HYGRO和SPEC的回收率低于50%; PRiME HLB是一种通用型的固相萃取柱,在净化蛋白质、磷脂等方面具有优越的性能,对10种氨基糖苷类药物的回收率均能达到70%以上。因此本研究选择PRiME HLB作为净化柱。

2.2.2 上样溶液pH的选择

氨基糖苷类药物属于碱性化合物,分子中含有多个氨基和羟基,每种组分具有多个p*K*_a_值,在不同的pH值条件下,以不同的离子或分子形式存在于溶液中,进而影响目标物在PRiME HLB上的保留。本研究考察了上样溶液pH值对10种氨基糖苷类药物回收率的影响(见图4)。结果表明,TOBRA、NEO、GENT受pH值影响较小,可以忽略不计;STREP、DHSTREP、SPEC、HYGRO在酸性条件下回收率较低,AMIK、KANA、APRA在碱性条件下回收率较低;在中性条件下,10种氨基糖苷的回收率最好。因此,上样溶液的pH值要控制在6~7。

**图4:**
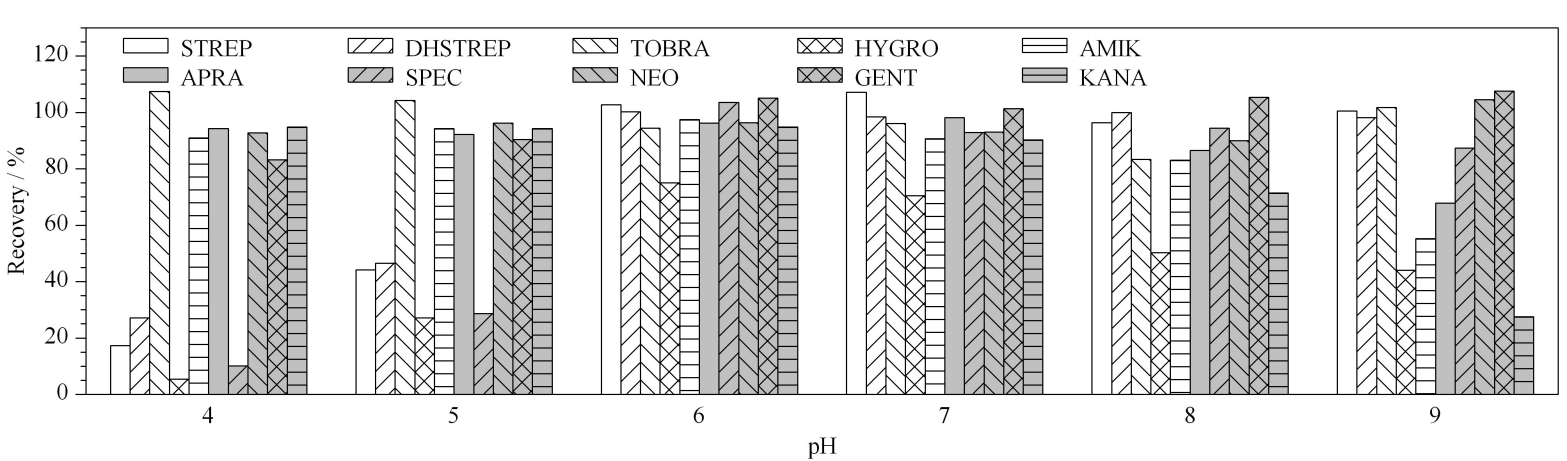
pH对鸡蛋中10种氨基糖苷类药物加标回收率的影响

### 2.3 色谱条件的优化

由于分子中氨基和羟基的存在,氨基糖苷类药物在传统的反相C_18_色谱柱上无保留,需要在流动相中加入七氟丁酸、全氟戊酸等离子对试剂,与氨基糖苷类药物结合形成疏水型离子对,从而改善峰形、增强保留,但离子对试剂不易挥发,对离子源有污染,影响质谱离子化效率,从而降低灵敏度。为了避免离子对试剂的使用,本研究选择亲水作用色谱和离子交换色谱来分析氨基糖苷类药物。

目前报道的氨基糖苷类药物分离的固定相有:氨基^[19]^、未改性的裸硅胶^[8]^、酰胺^[20]^、两性离子^[21]^和混合型两性离子^[19]^。本研究考察了未改性裸硅胶BEH HILIC(100 mm×2.1 mm, 1.7 μm, Waters公司)、酰胺键合的BEH Amide(100 mm×2.1 mm, 1.7 μm, Waters公司)和混合型两性离子键合的Obelisc R (150 mm×2.1 mm, 5 μm, SIELC) 3种类型的色谱柱。结果表明,Obelisc R色谱柱对10种氨基糖苷的分离最好,这归因于氨基糖苷类药物为碱性化合物,当pH<p*K*_a_时,目标物离子化,与SIELC Obelisc R键合的羧基相互作用,实现化合物的保留;在BEH Amide色谱柱上,由于酰氨基为中性基团,离子交换作用较小,亲水分配占主要地位,因此分离效果较差;在BEH HILIC色谱柱上,硅醇基与氨基糖苷类药物之间存在强氢键作用^[22]^和离子交换作用^[23]^,导致峰宽较宽,峰形较差。

### 2.4 质谱条件的优化

氨基糖苷类抗生素的结构中含有多个羟基,极性很强,因此选择电喷雾离子源。分别考察氨基糖苷类药物在正离子和负离子模式下的响应,发现正离子模式比负离子模式的响应高很多,这归因于氨基糖苷类物质的结构中含有多个伯胺或仲胺基团,具有弱碱性,易被电离成正离子。将标准溶液注入HPLC-MS/MS仪,在全扫描模式下,获得化合物的分子离子峰,结合各化合物的分子式,本研究选择的分子离子峰均为[M+H]^+^;在产物离子扫描模式下,以各个化合物的分子离子峰为母离子,进行二级质谱扫描,获得碎片离子,选择两对丰度较强,基质干扰较小的离子为子离子,优化后的质谱条件见表1。

### 2.5 基质效应

样品中除分析物以外的组分常常对分析物的离子化效率产生显著的干扰,影响分析结果的准确性,因此需要对基质效应进行评价。ME≈0,表明无明显的基质效应,若ME>0,表明基质效应增强,若ME<0,表明基质效应抑制。鸡蛋中10种氨基糖苷类药物的基质效应见图5,结果表明,STREP、AMIK、KANA基本无基质效应,DHSTREP、TOBRA、HYGRO、APRA、SPEC、NEO的|ME|小于30%, GENT基质效应强。因此,本研究采用空白基质溶液配制标准曲线,以消除基质效应的影响,提高分析结果的准确性。

**图5:**
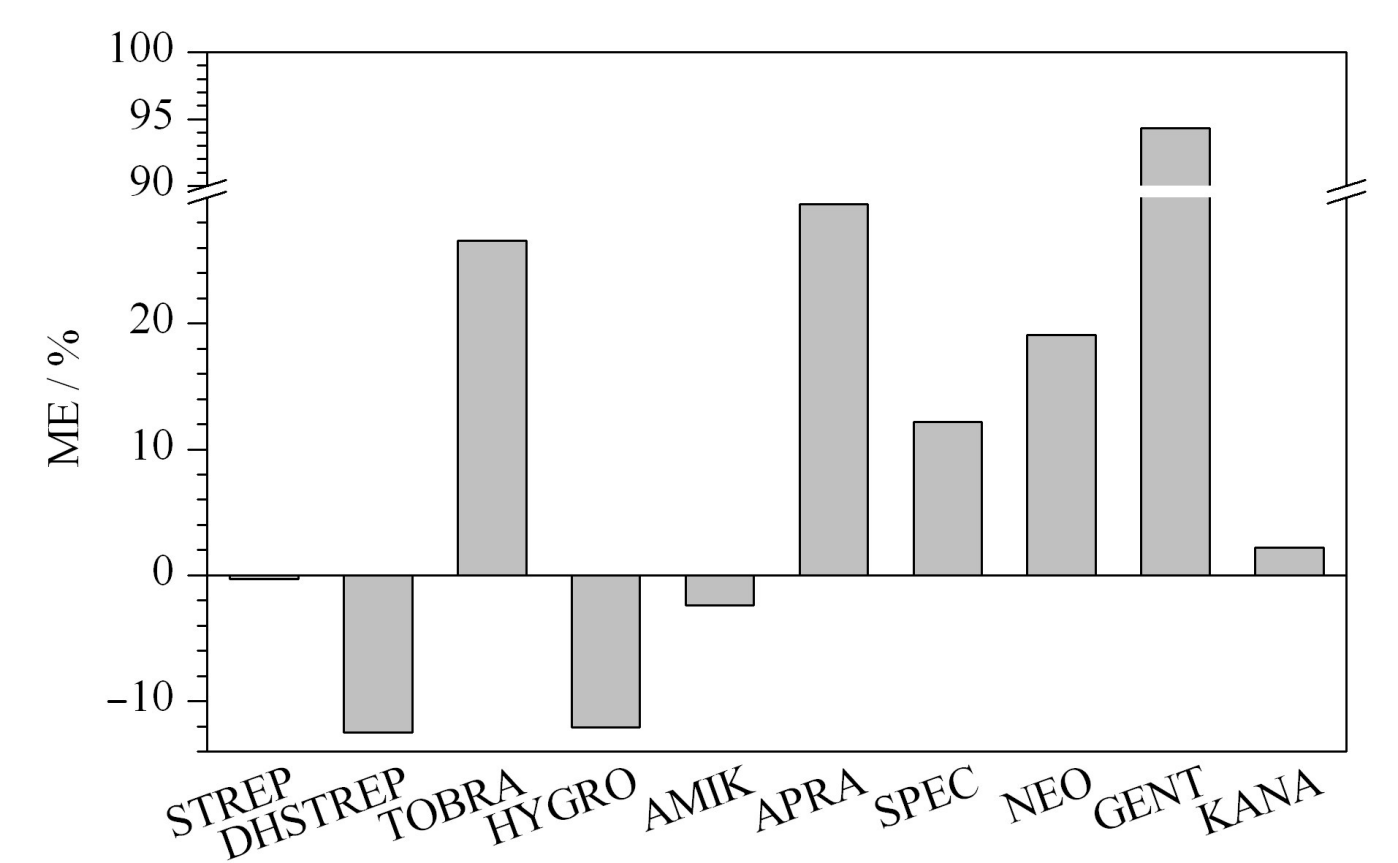
鸡蛋中10种氨基糖苷类药物基质效应的评价图

### 2.6 方法学验证

2.6.1 线性关系、检出限与定量限

将基质匹配标准工作溶液(5、10、20、50、100、200 μg/L)进行HPLC-MS/MS测定,获得标准溶液的质量色谱图。以定量离子的峰面积(*y*)对其质量浓度(*x*)作标准曲线,得到线性回归方程。分别向空白鸡蛋样品中加入2.5、5、12.5和25 ng的标准物质,以*S/N*≥3确定方法的检出限为2~5 μg/kg,以*S/N*≥10确定方法的定量限为5~10 μg/kg。10种氨基糖苷的线性范围、回归方程、线性相关系数、方法检出限和定量限见表2。在质量浓度5~200 μg/L范围内,定量离子的峰面积与质量浓度之间呈良好的线性关系,相关系数均大于0.99。空白鸡蛋中10种氨基糖苷类药物定量限水平的多反应监测色谱图见图6。

**表2:** 10种氨基糖苷类药物的线性范围、回归方程、相关系数、检出限及定量限

Compound	Linear equation	r^2^	LOD/ (μg/kg)	LOQ/ (μg/kg)
STREP	y=6702.12x-1056.57	0.9965	2	5
DHSTREP	y=23670.7x-5840.26	0.9984	2	5
TOBRA	y=7226.66x+3103.68	0.9970	2	5
HYGRO	y=5595.72x+5090.83	0.9931	2	5
AMIK	y=11406.5x+743.562	0.9974	2	5
APRA	y=5200.19x-3558.18	0.9951	2	5
SPEC	y=4500.79x+7301.67	0.9982	2	5
NEO	y=1795.86x+2724.18	0.9922	5	10
GENT	y=3699.14x+6061.60	0.9983	2	5
KANA	y=15051.8x+1638.55	0.9976	2	5

*y*: peak area; *x*: mass concentration, μg/L; linear range: 5-200 μg/L.

**图6:**
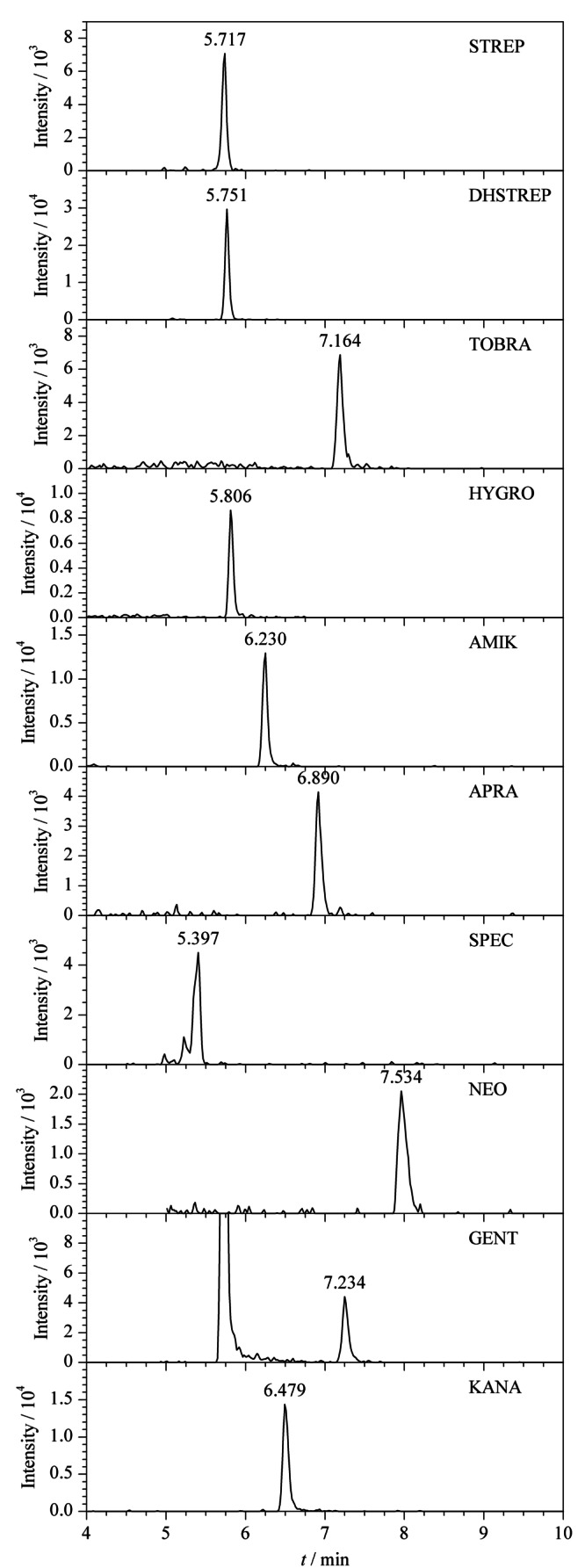
空白鸡蛋中10种氨基糖苷类药物定量限水平的多反应监测色谱图

2.6.2 回收率与精密度

分别向空白鸡蛋样品中添加低、中、高3个质量浓度水平的标准溶液,每个水平做6个平行实验,测定10种氨基糖苷类药物的回收率,考察方法的精密度(见表3)。由表3可知,鸡蛋样品中10种氨基糖苷类药物的加标回收率在68.1%~111.3%之间,RSD在1.2%~12.3%之间,方法的准确度和精密度满足GB/T 27404-2008《实验室质量控制规范食品理化检测》的要求。

**表3:** 10种氨基糖苷类药物在不同添加水平下的回收率和精密度(*n*=6)

Compound	LOQ level			20 μg/kg			100 μg/kg	
	Recovery/ %	RSD/ %		Recovery/ %	RSD/ %		Recovery/ %	RSD/ %
STREP	93.6	8.0		90.7	3.9		93.1	5.0
DHSTREP	100.7	5.4		93.3	2.5		95.2	2.1
TOBRA	94.4	8.7		94.0	6.8		104.8	4.9
HYGRO	68.1	8.0		68.9	3.7		72.0	3.8
AMIK	89.4	6.1		90.8	5.8		92.0	1.8
APRA	87.6	5.9		91.7	5.0		96.1	3.5
SPEC	73.3	8.0		84.2	4.0		93.5	1.2
NEO	87.0	9.9		91.2	7.9		99.5	5.3
GENT	86.2	12.3		97.5	11.1		111.3	6.8
KANA	89.5	6.2		90.4	4.4		93.3	1.9

### 2.7 实际样品分析

采用本方法对山东省各个超市采购的20批次鸡蛋样品进行测定,根据化合物的色谱保留时间和质谱离子丰度进行鉴别,所测样品中均未检出氨基糖苷类药物残留。

## 3 结论

本工作建立了鸡蛋中10种氨基糖苷残留量的高效液相色谱-串联质谱分析方法。该方法前处理流程操作简便,目标化合物获得良好的回收率,检测灵敏度高,重现性好,满足方法确认要求,适用于鸡蛋中氨基糖苷类药物残留的批量检测。
